# MRI-based radiotherapy planning method using rigid image registration technique combined with outer body correction scheme: a feasibility study

**DOI:** 10.18632/oncotarget.17672

**Published:** 2017-05-07

**Authors:** Ki Mun Kang, Hoon Sik Choi, Bae Kwon Jeong, Jin Ho Song, In-Bong Ha, Yun Hee Lee, Chul Hang Kim, Hojin Jeong

**Affiliations:** ^1^ Department of Radiation Oncology, Gyeongsang National University School of Medicine and Gyeongsang National University Hospital, Jinju, Republic of Korea; ^2^ Institute of Health Sciences, Gyeongsang National University, Jinju, Republic of Korea; ^3^ Department of Radiation Oncology, Gyeongsang National University Changwon Hospital, Changwon, Republic of Korea

**Keywords:** MRI-based radiotherapy, rigid image registration, brain tumor, radiotherapy planning

## Abstract

An alternative pseudo CT generation method for magnetic resonance image (MRI)-based radiotherapy planning was investigated in the work. A pseudo CT was initially generated using the rigid image registration between the planning MRI and previously acquired diagnostic CT scan. The pseudo CT generated was then refined to have the same morphology with that of the referenced planning image scan by applying the outer body correction scheme. This method was applied to some sample of brain image data and the feasibility of the method was assessed by comparing dosimetry results with those from the current gold standard CT-based calculations. Validation showed that nearly the entire pixel doses calculated from pseudo CT were agreed well with those from actual planning CT within 2% in dosimetric and 1mm in geometric uncertainty ranges. The results demonstrated that the method suggested in the study was sufficiently accurate, and thus could be applicable to MRI-based brain radiotherapy planning.

## INTRODUCTION

Since the development of three-dimensional conformal radiotherapy, volumetric image data have been routinely used in radiotherapy planning to compute volume dose distribution [1–5]. The essential requirements of volumetric image for radiotherapy planning include geometric accuracy [3], anatomic visibility [3], production of set-up and verification images [4], and the determination of mass-electron density to estimate interactions between radiation and matter [2, 5].

Computed tomography (CT) scan is considered the gold standard image for radiotherapy planning, as it fulfills (fully or almost fully) the requirements described above [1–5]. However, because CT has relatively low soft-tissue contrast, efforts have been made to replace CT with magnetic resonance images (MRI) for radiotherapy planning [6–11], because MRI has better soft-tissue contrast than CT [12].

Several core technology issues for MRI-based planning have been developed and improved. These include the correction for geometric distortion of MRI [13], the development of MRI-based in-treatment room imaging for set-up and positioning verification [14], and the integration of the MRI system with a radiotherapeutic unit [15, 16]. However, a concern still remains regarding to the determination of the mass-electron density for exact dose distribution calculation, which is directly determinable with CT [2, 5], but not yet with MRI. Previous studies indeed showed that no appropriate correction for mass-electron density could yield substantial calculation error even in the relatively homogeneous brain region [8].

Several approaches have been proposed to synthesize CT-like images (referred as pseudo CT) from MRI to provide mass-electron density [6–11]. The most straightforward method is to segment MRI into several partitions, each having similar mass-electron density, and to assign representative CT values in to them [6–8]. This method, denoted as the segmentation and assigning CT number method (SAC), is generally successful in most of clinical situation, but requires a large workload for manual segmentation.

An alternative approach is the direct conversion of MRI signals to CT numbers (DCMC) using advanced MRI sequences, such as ultrashort echo time sequences [6, 9, 10]. Although this approach has shown promising results, there is a practical concern regarding the need for extra-MRI acquisitions far beyond the routine.

Another approach is based on the atlas-based deformable registration (ADR) technique [6, 11], in which CT numbers are transferred into MRI from the pre-stored CT/MRI data set via a deformable registration technique. This method is probably more efficient than the SAC and DCMC methods, because no extra workload is required for structural segmentations and no extra MRI sequences beyond the routine are needed. However, potential registration errors, particularly when applied to a patient with atypical anatomic shape, as well as the large computation time required to minimize inter-patient registration errors, remain unresolved

Here we suggested a new alternative by employing the rigid image registration technique combined with manual outer body correction scheme. We applied the method to some brain MRI and investigated the feasibility of the method by comparing the dosimetry results with those obtained from the current golden standard CT-based calculations. The method was sufficiently accurate, as well as being more time and cost efficient than other previous methods, demonstrating its practical feasibility in radiotherapy planning for brain tumors.

## RESULTS

### Time efficiency

Although not explicitly measured, the present pseudo-CT generation method was sufficiently time efficient, as all CPU processes for each preparation step, including rigid registration, grid interpolation, and auto body contour, were almost instantly completed within about 10 seconds. The net CPU time to complete the entire processes was ≤ 30 seconds. Although additional manual software operation time was needed, such as for copying and pasting body contours and manually assigning CT density values, the total image preparation for each patient did not take over 10 minutes, even when the time for manual operation was included.

### Original plan quality

The original plans constructed on the pseudo-CT met the desired requirements well, as summarized in Table 1. The 95% and 100% prescribed-dose surfaces covered almost 100% (the least V_95%_ = 99.8%) and exactly 95% of the target volumes, respectively. The uniformity of target dose distribution was sufficient, as the Max PTV doses were not higher than 110%, while the Min PTV were mostly higher than 95% of prescription dose. The dose conformity was also good as the conformity index (1.07 ± 0.04, range: 1.01–1.17) was only little higher than the ideal of 1.0.

**Table 1:** Dosimetric quantities in the original plans and their variations (Δ) in the recalculated verification plans, where maximal (Max), mean, minimal (Min) PTV doses, PTV coverage (V100%), and the conformity index at the 100% prescription dose are given

Group	Ord		Vent		Skull		Over all	
	Original	Δ	Original	Δ	Original	Δ	Original	Δ
Max	105.1 ± 1.3 (103.0–107.5)	0.0 ± 0.4 (−0.4–0.8)	105.4 ± 1.1 (103.2–106.5)	0.0 ± 0.2 (−0.3–0.6)	107.0 ± 2.0 (103.5–110.1)	−0.2 ± 0.4 (−1.2–0.4)	105.8 ± 1.7 (103.0–110.1)	0.0 ± 0.4 (−1.2–0.8)
Mean	102.2 ± 0.4 (101.4–102.9)	0.0 ± 0.2 (−0.3–0.3)	102.0 ± 0.4 (101.2–102.9)	0.0 ± 0.2 (−0.3–0.3)	102.5 ± 0.8 (101.5–104.0)	0.0 ± 0.2 (−0.3–0.5)	102.2 ± 0.6 (101.2–104.0)	0.0 ± 0.2 (−0.3–0.5)
Min	95.8 ± 2.0 (92.2–98.5)	0.0 ± 0.3 (−0.5–0.5)	95.0 ± 2.5 (90.2–97.5)	0.0 ± 0.3 (−0.5–0.3)	94.4 ± 3.3 (87.1–98.9)	−0.6 ± 1.3 (−4.0–0.7)	95.1 ± 2.6 (87.1–98.9)	−0.2 ± 0.8 (−4.0–0.7)
V95%	100.0 ± 0.0 (99.9–100)	0.0 ± 0.0	100.0 ± 0.0	0.0 ± 0.0	100.0 ± 0.1 (99.9–100.0)	0.0 ± 0.2 (−0.2–0.0)	100.0 ± 0.0 (99.8–100.0)	0.0 ± 0.0 (−0.2–0.0)
V100%	95.0 ± 0.0	−0.4 ± 0.9 (−1.8–1.5)	95.0 ± 0.0	−0.2 ± 1.3 (−1.9–1.9)	95.0 ± 0.0	−0.4 ± 0.9 (−1.8–1.5)	95.0 ± 0.0	−0.3 ± 1.0 (−1.9–1.9)
CI	1.07 ± 0.04 (1.02–1.15)	−0.3 ± 1.0 (−1.9–1.9)	1.02 ± 0.02 (1.01–1.06)	−0.2 ± 0.4 (−1.1–0.4)	1.08 ± 0.04 (1.04–1.17)	−0.2 ± 1.3 (−1.9–1.9)	1.07 ± 0.04 (1.01–1.17)	−0.2 ± 1.0 (−1.9–1.9)

The data are given in means ± standard deviations with the ranges shown in parentheses.

Plan quantity indicators of target coverage, uniformity, and conformity did not differ among the plan groups for the three different PTV of PTV_ord_, PTV_vent_, and PTV_skull_ (Table 1), indicating that the quality of the original treatment plans was independent of tumor site.

### Dosimetric changes in verification plans

The dosimetric quantities were almost unchanged when the plans were recalculated, after replacing volumetric image data from pseudo CT with PCT. As shown in Table 1, the Max, mean, and Min PTV doses changed < 2% in all but one plan, with the latter showing a decrease in Min PTV dose more than 2% (**−**4%). Other dosimetric quantities for CI and target coverages were not changed by more than 2% without exception (Table 1). The magnitudes of changes in the dosimetric quantities were very similar among the three different plan groups as summarized in the Table 1.

**Figure 1:**
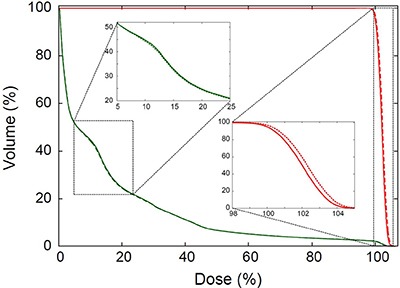
Dose-volume histogram (DVH) for the plan that showed the largest difference in the minimal PTV dose between the original and verification plans The DVH for body (green) and PTV (red) in the original (solid lines) and verification plans (dotted lines) are plotted. The insets are the enlarged views for the dotted rectangles in the figure.

In Figure 1, a dose-volume histogram for a plan showing the largest Min PTV dose change after recalculation is displayed, for reference.

### Pixel-to-pixel dose agreement

**Figure 2:**
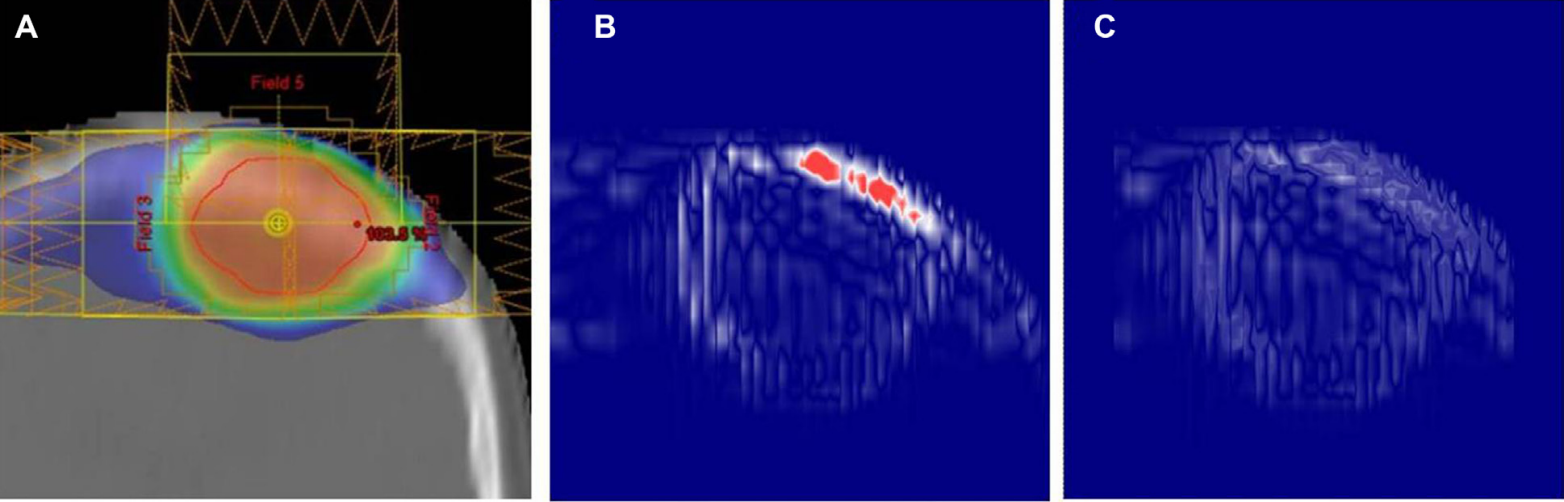
Center-coronal dose analysis results for the worst pixel-dose agreement case: **(A**) dose distribution in the original plan, (**B**) pixel-to-pixel dose difference map, and (**C**) gamma agreement map between the original and verification plans, respectively, with 2% in dosimetric and 1% in geometric acceptance criteria. In (B), the pixels having the dose differences higher than 2% are shown in red color.

The pixel-to-pixel agreement in dose distributions between the original and recalculated verification plans was assessed on the 10 × 10 cm^2^ cubic plane centered at each plan iso-center. When evaluating the agreement based on the 2% dose difference criterion, 99.86 ± 0.28% of pixels were in agreement with each other (Table 2). Only a small rate of pixels (less than 1.20% at the most) showed the difference larger than 2% with maximal difference up to 14.38%. The agreement was similar over all three different plan groups, with Skull group showing slightly higher disagreement (passing rate: 99.66 ± 0.42%) compared than the other two Ordinary (99.97 ± 0.05%) and Ventricle (99.95 ± 0.08%) groups. The poorest agreement was also observed in the Skull group, in which 98.8% of pixels satisfying the < 2% dose difference criterion. The dose difference map for this worst agreement case is displayed in Figure 2, along with the dose distribution map in the original treatment plan.

**Table 2:** Pixel-to-pixel dose differences in absolute value (|Δ|) and gamma (γ) agreements with 2% in dosimetric and 1 mm in geometric acceptance criteria, respectively, where the mean, standard deviation (σ), maximal deviations (Max), and passing rate within the acceptance criteria for each agreement results are given

Data	Quantity	Ord	Vent	Skull	Over all
|Δ|	Mean	0.06 ± 0.04 (0.02–0.15)	0.08 ± 0.04 (0.03–0.16)	0.08 ± 0.05 (0.03–0.18)	0.07 ± 0.04 (0.02–0.18)
	σ	0.12 ± 0.06 (0.06–0.23)	0.34 ± 0.42 (0.05–1.13)	0.22 ± 0.10 (0.07–0.38)	0.22 ± 0.26 (0.05–1.13)
	Max	2.50 ± 2.83 (0.45–9.78)	3.44 ± 4.97 (0.21–14.38)	4.26 ± 3.41 (0.70–12.87)	3.40 ± 3.79 (0.21–14.38)
	rate	99.97 ± 0.05 (99.85–100)	99.95 ± 0.08 (99.82–100)	99.66 ± 0.42 (98.80–100)	99.86 ± 0.28 (98.80–100)
γ	Mean	0.03 ± 0.02 (0.01–0.06)	0.03 ± 0.02 (0.01–0.08)	0.03 ± 0.02 (0.01–0.08)	0.03 ± 0.02 (0.01–0.08)
	σ	0.04 ± 0.02 (0.02–0.08)	0.04 ± 0.02 (0.01–0.07)	0.06 ± 0.02 (0.03–0.10)	0.05 ± 0.02 (0.01–0.10)
	Max	0.40 ± 0.20 (0.23–0.96)	0.28 ± 0.14 (0.10–0.51)	0.70 ± 0.44 (0.28–1.96)	0.46 ± 0.34 (0.10–1.96)
	rate	100.0 ± 0.0	100.0 ± 0.0	99.99 ± 0.03 (99.89–100)	˜100.0 ± 0.02 (99.89–100)

All data are given in means ± standard deviations with the ranges shown in parentheses.

**Figure 3:**
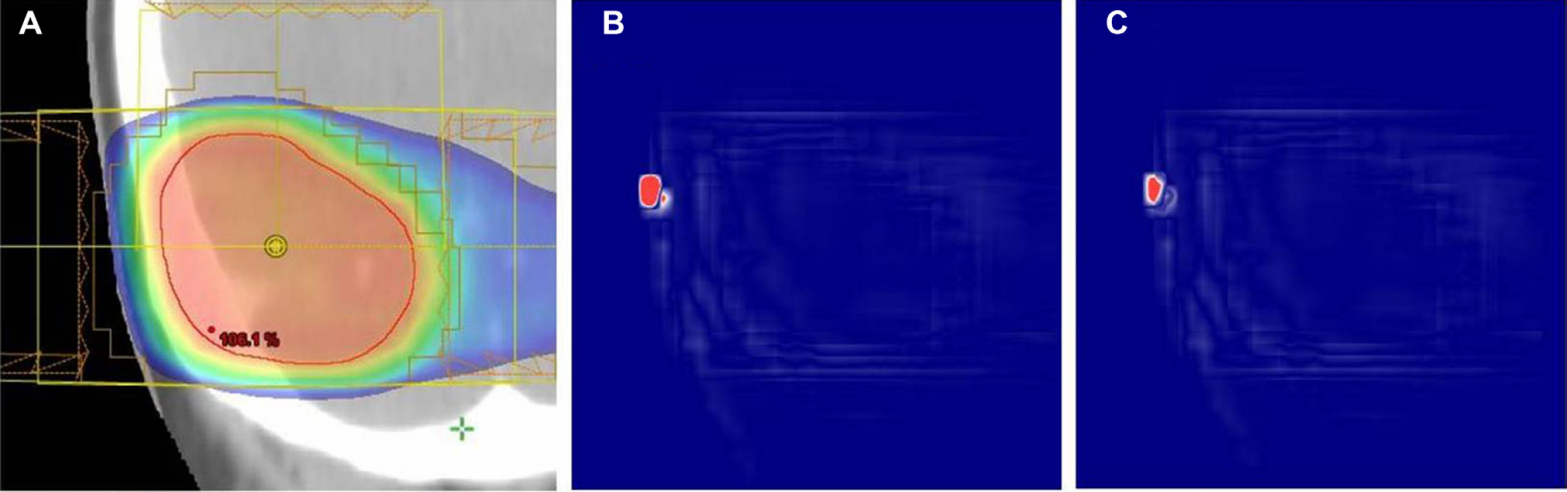
Center-coronal dose analysis results for the worst gamma agreement case: (**A**) dose distribution in the original plan, (**B**) pixel-to-pixel dose difference map, and (**C**) gamma agreement map between the original and verification plans, respectively. In (B) and (C), the pixels that failed the dose (2%) and gamma (2%/1 mm) difference criteria are shown in red color.

The gamma (γ) agreements between the original and verification plans were calculated based on the 2%/1 mm acceptance criteria. The gamma passing rates and the pixel population rates in which γ ≤ 1.0 were perfect in 32 of 33 plans. Only one plan, which is not the same case with the worst pixel-dose agreement case described above, in the skull group did not show complete gamma agreement. However, even in this worst agreement plan, agreement was slightly less than perfect (99.89%) with the maximum gamma of 1.96. The overall mean gamma passing rate for the 33 plans was almost perfect (˜100 ± 0.02%). The gamma agreement map for the worst case is also displayed in Figure 3, for reference.

## DISCUSSION

This study investigated the feasibility of an MRI-based planning method for brain radiotherapy using the rigid registration of pre-taken diagnostic CT to planning MRI, focusing on planning efficiency and dosimetric accuracy. This method was applied to data from 11 actual brain tumor patients. Validation showed that this method was more efficient than any other MRI-based planning methods developed to date [7–11].

Other methods, including SAC [7, 8] and DCMC [9, 10] methods, are either time-consuming, requiring structural segmentation (SAC), or are cost-consuming, requiring additional MRI sequences (DCMC). These steps, however, are not needed in our method. Although our method also requires external body contouring, the latter is any way needed for radiotherapy planning, indicating that it does not constitute an extra workload.

The present method is also more efficient than the ADR method [11], which uses data from different patients to derive pseudo-CT images. In contrast, the present method uses data from the same patients. Therefore, the ADR method essentially requires a deformable matching process to correct for inter-patient anatomic differences. This requires additional computation time and a higher-performance computation environment than the rigid image registration used in our method. Furthermore, the single application of deformable registration is generally not sufficient, but must be repeated with multiple patient data sets to sufficiently eliminate inter-patient registration errors. The average CPU time required for pseudo CT generation was reported to be ˜16 minutes when using a single atlas data and to be ˜270 minutes when using 12 number of multiple atlas data sets [11].

**Figure 4:**
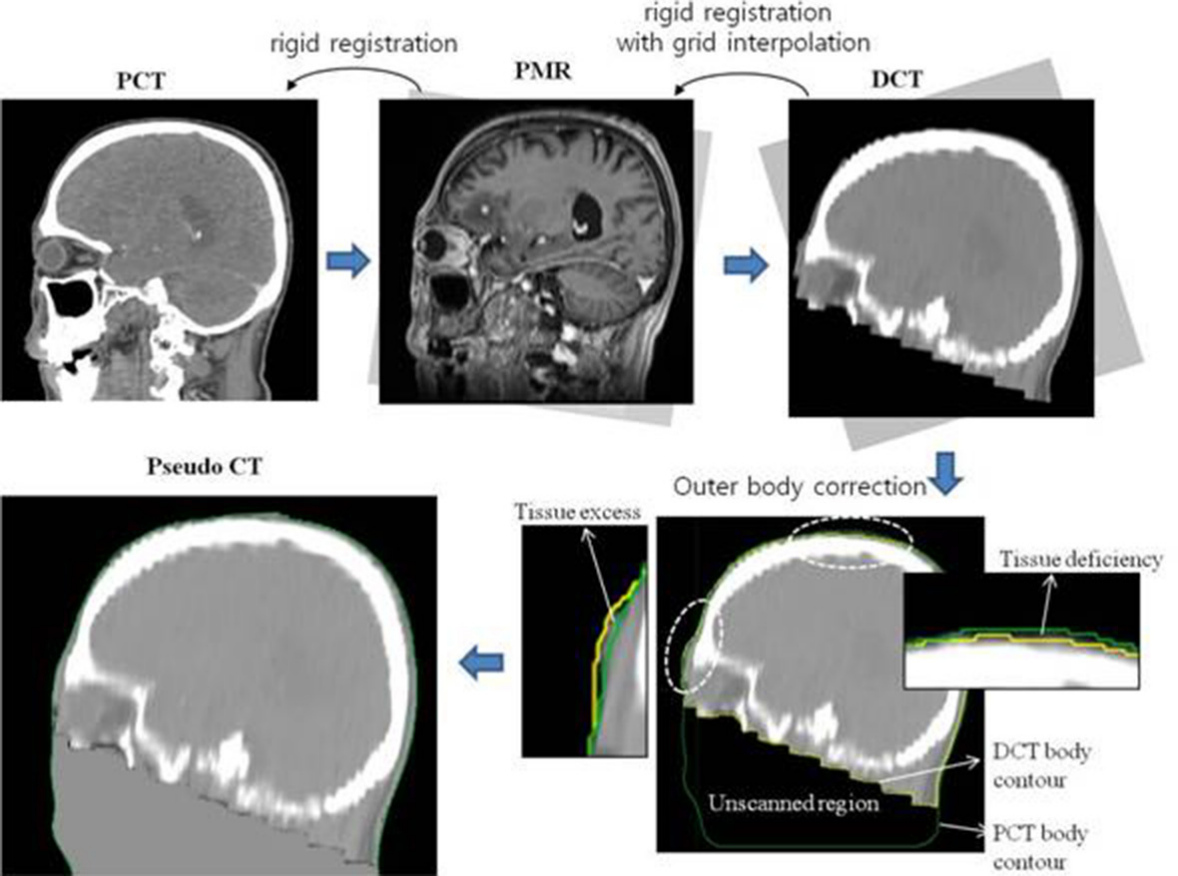
Work flow for pseudo-CT generation method used in the study

Inter-image differences may arise, even when using the same patient's scan set. This error should be also corrected for exact dose calculation, but the correction cannot be made with the rigid image registration technique. To simplify this problem, we assumed that all inter-image differences between brain scans occur in the outer skull region, whereas internal anatomic changes would be negligible for adult patients. Using this assumption, the inter-image differences for the outer skull region were simply corrected by matching the outer body alone, as schematically displayed in Figure 4.

**Figure 5:**
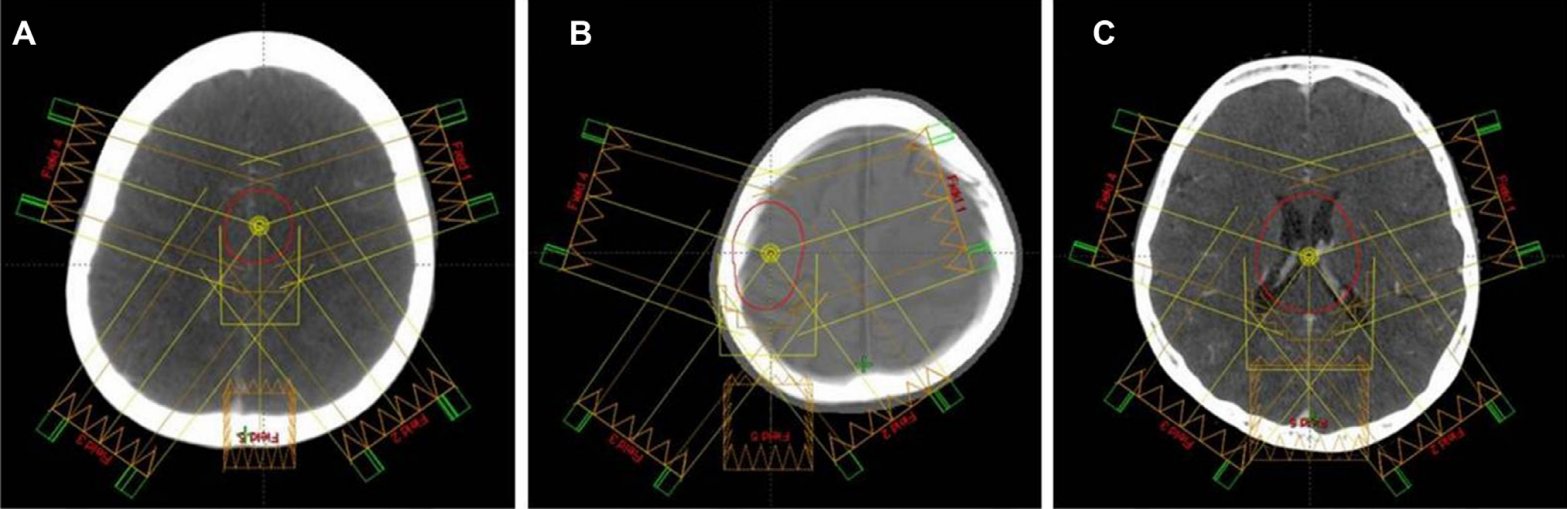
Typical target volumes delineated on pseudo-CT scans: (**A**) PTV_ord_, (**B**) PTV_skull_, and (**C**) PTV_vent_. In each figure the field alignment in each treatment plan is shown.

Although the correction was only an incomplete approximation, it did not reduce dosimetric accuracy. As shown in Figure 5 and Table 2, all the dosimetric quantities calculated on the MRI-derived pseudo CT scans, including Min, mean, Max PTV doses, and dose volume histograms were almost same within 2% in dosimetric acceptance criteria when compared with the results of CT-based calculations. In addition, the pixel-to-pixel dose agreement results met well the dosimetric acceptance criteria in most of pixels in the pseudo CT scans used in the study (passing rate: 99.86 ± 0.28%) as summarized in Table 3. Although some tiny rate of pixels failed to meet the criteria with maximum deviation up to 14.38%, these disagreement pixels were completely eliminated in all the plans with only one exception (shown in Figure 3) when reanalyzing the agreements with allowing a 1-mm geometric error along with the 2% dosimetric error, i.e., gamma agreement. This demonstrated that the registration error in the method due to inter-image differences between DCT and PMR(PCT) may be acceptable within 2%/1mm uncertainty ranges. It is also notable that the dose agreements between the original and verification plans were almost the same wherever tumors were located, from the lowest density (ventricle) to highest density (skull) regions in the brain (Table 2). According to the previous MRI-based planning study based on the SAC method, it was reported that no consideration of tissue heterogeneity substantially overestimated the target coverage in brain lesion up to ˜16% [8]. The effect of tissue heterogeneity correction was rechecked here using one of our patients’ CT data and found that non-negligible dose differences (˜9.0% in maximal pixel dose and ˜2.0% in mean tumor dose differences) could take place only by the tissue heterogeneity effect. Considering the calculation results as well as the sensitive dependence of the AAA algorithm on tissue heterogeneity [19], the region-wide agreement in dose observed in the present study may imply the reliable mass-electron density mapping of MRI-derived pseudo CT scans.

**Table 3:** Characteristics of patients and target volumes delineated

Characteristics	Mean ± standard deviation	Range
Age (years)	59.1 ± 11.6	40.0–75.0
Sex	6 male, 5 female	
Scan time interval (months)	3.7 ± 4.9	0.1–13.1
Tilted gantry angle in DCT	13.6 ± 7.4°	0.0°–3.3°
Target Volume (cc)		
PTV_ord_	10.5 ± 9.4	1.2–33.8
PTV_vent_	51.7 ± 20.0	28.7–97.0
PTV_skull_	26.5 ± 12.1	8.9–19.35

The ages are given referenced to the time of planning CT(MR) scanning and the scan time interval indicates the time interval between DCT and PCT scanning.

The present MRI-based planning method, although highly successful in the patients examined in this study, may have several limitations. The first limitation is that it may only be applicable to patients who underwent at least one previous CT scan before radiotherapy. At present, however, CT is the primary imaging modality in the diagnosis of brain tumors, suggesting that this limitation may not be a problem to most patients requiring radiotherapy. Although MRI may be better diagnostically, is remains a secondary diagnostic modality because of its higher cost and lower penetration rate than CT. The second limitation is its limited applicability to tumors in rigid organs, such as brain, head and neck, and extremity. The third limitation is that the applicability of the method would be dependent on the quality of the pre-taken CT scan. However, this problem would not be critical in most cases as it was highly successful with the present diagnostic CT data, despite these CT data being non-ideal for planning, with wide slice thickness (5 mm) and with non-axial-parallel orientation (titled gantry angle range: 0–23.3°).

## MATERIALS AND METHODS

### Patient selection

Eleven patients with fully-grown adult brain tumors, who had undergone at least one diagnostic CT (denoted as DCT) scan before radiotherapy, and had undergone planning CT (PCT) and MRI (PMR) scans for brain radiotherapy, and who had not undergone brain surgery during the period of DCT and PCT (PMR) scanning, were included in this study.

DCT scans for each patient were acquired with dual pre- and post-contrast enhancements and with 5-mm slice thickness using the Ingenuity CT scanner (manufactured by Philips Healthcare System) with tilted gantry angles. The tilt angles were individually optimized to exclude radiosensitive lenses while including the entire cranium in the CT scan region [17], which ranged from 0° to 23.3° for individual patients. All the DCT scans were acquired prior to PCT/PMR scanning for brain radiosurgery planning, with the time intervals between the scans ranging from 3 days to 13.1 months (Table 3).

The PCT and PMR scans for individuals were acquired using the LightSpeed Plus CT scanner (GE Healthcare Inc., Milwaukee, Wis., USA) and 1.5T Magnetom Avanto MRI system (Siemens Medical Solutions, Erlangen, Germany), respectively. Both scans were taken on the same day while retaining the same set-up position. These scans ranged from the cranial vertex at least to the second cervical spine level. No tilt angle was applied during PMR or PCT scanning. The slice thicknesses were 1.25 mm for PCT scans and 2.5 mm for PMR scans. The PCT for each patient was single contrast enhanced, and the PMR for each patient was dual T1- and T2-weighted enhanced.

### Post imaging process

For actual MRI-based radiotherapy, the planning MRI scan should be set as the primary image scan for planning, and therefore must be kept fixed during the entire planning procedure [6]. However, in this study, as detailed described below, the PCT, rather than the PMR, scan was fixed, while the PMR was allowed to be reoriented along the anatomic orientation of the PCT scan. This reorientation was allowed only to quantitatively compare the dosimetric accuracy of the present pseudo-CT method with the gold standard planning-CT based calculations. This comparison requires the pseudo CT and planning CT scans to have the same geometric dimensions and anatomic orientations.

After acquiring the dual pre- and post-contrast DCT and T1- and T2-weighted PMR scans for each patient, only the post-contrast DCT and T1-weighted PMR scans were chosen for post-image processing. Post-image processing for pseudo-CT generation was performed using the HP workstation equipped with Intel i5 CPU, and MiM maestro software (MiM Software, Cleveland, OH, USA).

The step-by-step flow for image processing is described below and schematically illustrated in Figure 4. First, the PMR scan was rigidly fused or reoriented with respect to the PCT scan until the bony structures of the skull on the two scans were best matched. Second, the rigid registration of DCT scan was applied to the reoriented PMR scan. Third, the reoriented DCT scan was interpolated to have the same grid size as the PMR scan. Fourth, the external body contours of the DCT and PCT scans were delineated based on CT pixel density. Finally, the tissue excesses and deficiencies of the DCT scan with respect to the referenced PCT scan were corrected so that both scans had the same outer body shape. In detail, the pixel regions in the DCT scan that were located outside the PCT body contour were defined as the tissue-excessive regions and their pixel CT values were corrected to the air-equivalent value (-1000 HU). Regions within the PCT body contour but outside the DCT body contour were defined as the tissue-deficient regions and their CT values were assigned the soft tissue-equivalent value (0 HU). Some unscanned tissues in the ordinary DCT scan (see Figure 4) were also included as tissue deficiencies and corrected to 0 HU with no consideration of their real CT electron density.

Once the above steps are complete, the DCT scan has the same skull orientation as the PCT (and also reoriented PMR) scan, and has the same outer body shape as the PCT scan. Corrected DCT scans are described as post-generated pseudo CT scans and used here to calculate 3D dose distributions.

The procedures described above must be modified slightly when applied to actual MRI-based planning. The first step, the rigid fusion of PMR relative to PCT scans, should be skipped and the manual outer body correction described in the fourth and fifth steps should be applied relative to the external body for PMR, rather than PCT, because all geometric information must be referenced from MRI scans for actual MRI-based planning.

### Target contours

Three different planning tumor volumes (PTV) for individuals were delineated on the fused PMR and post-generated pseudo CT scan sets. The first PTV was delineated at the ordinary tumor sites of individual patients (hereafter denoted PTV_ord_). The other two targets were pseudo targets and delineated near the highest- (skull) and least-density (ventricle) structures in the brain, in order to investigate the effect of tissue heterogeneity on calculations of dose distribution [18]. These two pseudo targets are denoted PTV_skull_ and PTV_vent_, respectively.

Figure 5 shows a typical example for each delineated PTV, and Table 3 summarizes the detailed characteristics of PTV delineated.

### Treatment planning

All the treatment plans for each patient, based on three different tumor volumes, were initially created on post-generated pseudo CT scans. The heterogeneity-sensitive analytical anisotropic algorithm (AAA) [19], incorporated in the Eclipse treatment planning System version 10.0 (Varian Medical System, Palo Alto, CA, USA), was used in all planning processes. The plans were optimized using the five intensity-modulated photon beams, where four of five beams were aligned on the coplanar and the remaining beam aligned on non-coplanar planes (Figure 5). All the treatment beams were aligned so that they did not enter through the unscanned region marked in Figure 4, because in which the assigned CT values were not exact.

The individual patient plans were optimized to meet the following criteria: i) 95% and ii) 100% of the prescribed dose surfaces should cover 100% and 95%, respectively, of the target volumes; (iii) the maximum dose in PTV should be no higher than 110% of the prescribed dose, and iv) the dose conformity index, defined as the ratio between the PTV and the irradiation volume receiving doses higher than the prescribed dose [20], should be as close as possible to 1.00 and no higher than 1.20.

Once optimized, the original treatment plans were recalculated using the PCT scans, while all other planning parameters, as well as the optimized beam intensity profiles, were kept the same. The recalculated plans on the PCT scans will be denoted as the verification plans.

### Feasibility evaluation

The feasibility of the post-generated pseudo CT for use in radiotherapy planning was evaluated by comparing dose distributions by the original and verification plans. By benchmarking the high-end criteria in AAPM TG-142 for quality assurance of medical accelerators [21], acceptable feasibility was defined as pixel doses between plans < 2% in dosimetric and <1 mm in geometric uncertainty ranges, i.e., gamma agreement with 2%/1 mm criteria [22]. In addition, the general plan-quality indicators were compared for the original and verification plans; these indicators included minimum (Min), mean, and maximum (Max) PTV doses, dose conformity index (CI), and target coverages at 95% (V_95%_) and 100% (V_100%_) prescribed dose levels, respectively.

## CONCLUSIONS

This study describes the development of a simple but effective MRI-based planning method for brain radiotherapy. This method, which employs a rigid image registration technique combined with a manual outer body correction scheme, yielded fairly efficient and accurate results in planning for brain tumors. Thus, the method may be considered an alternative MRI-based planning method for the treatment of brain tumors.
